# IL-17a and IL-22 Induce Expression of Antimicrobials in Gastrointestinal Epithelial Cells and May Contribute to Epithelial Cell Defense against *Helicobacter pylori*

**DOI:** 10.1371/journal.pone.0148514

**Published:** 2016-02-11

**Authors:** Beverly R. E. A. Dixon, Jana N. Radin, M. Blanca Piazuelo, Diana C. Contreras, Holly M. Scott Algood

**Affiliations:** 1 Veterans Affairs Tennessee Valley Healthcare Services, Nashville, Tennessee, United States of America; 2 Department of Medicine, Vanderbilt University, Nashville, Tennessee, United States of America; 3 Department of Pathology, Microbiology and Immunology, Vanderbilt University, Nashville, Tennessee, United States of America; Instituto Butantan, BRAZIL

## Abstract

*Helicobacter pylori* colonization of the human stomach can lead to adverse clinical outcomes including gastritis, peptic ulcers, or gastric cancer. Current data suggest that in addition to bacterial virulence factors, the magnitude and types of immune responses influence the outcome of colonization. Specifically, CD4+ T cell responses impact the pathology elicited in response to *H*. *pylori*. Because gastritis is believed to be the initiating host response to more detrimental pathological outcomes, there has been a significant interest in pro-inflammatory T cell cytokines, including the cytokines produced by T helper 17 cells. Th17 cells produce IL-17A, IL-17F, IL-21 and IL-22. While these cytokines have been linked to inflammation, IL-17A and IL-22 are also associated with anti-microbial responses and control of bacterial colonization. The goal of this research was to determine the role of IL-22 in activation of antimicrobial responses in models of *H*. *pylori* infection using human gastric epithelial cell lines and the mouse model of *H*. *pylori* infection. Our data indicate that IL-17A and IL-22 work synergistically to induce antimicrobials and chemokines such as IL-8, components of calprotectin (CP), lipocalin (LCN) and some β-defensins in both human and primary mouse gastric epithelial cells (GEC) and gastroids. Moreover, IL-22 and IL-17A-activated GECs were capable of inhibiting growth of *H*. *pylori in vitro*. While antimicrobials were activated by IL-17A and IL-22 *in vitro*, using a mouse model of *H*. *pylori* infection, the data herein indicate that IL-22 deficiency alone does not render mice more susceptible to infection, change their antimicrobial gene transcription, or significantly change their inflammatory response.

## Introduction

*Helicobacter pylori* is a Gram-negative microaerophilic curved rod bacterium that subsists extracellularly in the gastric mucosa. Infection with *Helicobacter pylori* can lead to a number of disease outcomes including gastritis, peptic ulcer disease, gastric adenocarcinoma, or MALT lymphoma [1, 2]. Interestingly, disease outcomes are influenced by bacterial, environmental and host factors. The bacterial factors that can give rise to tissue damage include the Cag pathogenicity island that encodes a type IV secretion system (T4SS), adherence factors, and the isotype of the vacuolating toxin (VacA) [3–5]. These factors suggest that *H*. *pylori* strain variation significantly influences disease outcome. Environmental factors, such as diet, may also affect disease outcome indirectly by affecting expression of bacterial proteins, or by influencing the host immune response [6–9]. Finally, the host’s immune response plays a role in the control of infection and inflammation and thereby influences disease outcome.

The host immune response to *H*. *pylori* infection involves both cellular and humoral immunity along with an ongoing innate response including recruitment and activation of neutrophils and macrophages. The adaptive response is driven by both T and B cell responses. The T cell response is a mixed Th1-Th17-Th2 response [10–21], but the relative contributions of each of these subsets is still under investigation. There appears to be interplay between the Th1 and Th17 responses during *H*. *pylori* infection making it difficult to determine the relative contributions of these subtypes [22]. Th17 cells produce IL-17A, IL-17F, IL-21 and IL-22. While several studies have provided evidence that IL-17A contributes to innate immune cell recruitment [23–25], IL-17A and IL-22 are also associated with anti-microbial responses and control of bacterial colonization in several models. The roles of IL-17A and IL-22 in control of *H*. *pylori* proliferation has not been fully elucidated. Studies in IL-17RA-/- mice suggest that IL-17A signaling is required for control of bacterial burden [24]. Studies in IL-21-/- mice demonstrate that IL-21 is required for activation of Th1 and Th17 responses and therefore, demonstrates that IL-21 is required for control of bacterial colonization [26]. But, the contribution of IL-22 to bacterial colonization has not been elucidated.

IL-22 is produced by immune cells, including T-helper (Th) cell subsets and innate lymphocytes. Expression of IL-22 has been reported in the *H*. *pylori*-infected gastric tissue of humans [27] and gastric cancer patients [28, 29]. Recent studies have suggested that IL-22 targets cells of the digestive, respiratory, and skin organ systems, and plays an important role in host mucosal defense against some Gram-negative bacteria including *Klebsiella* and *Citrobacter rodentium* and the pathogenic yeast, *Cryptococcus neoformans* [30–34]. IL-22 is a member of the IL-10 cytokine family [35]. It can be expressed and secreted by a number of T cell subsets including Th1, Th17, γδ T cells, NK T cells, and the recently described Th22 cells [32, 36]. While it acts synergistically with a number of cytokines including TNF, IL-1β and IL-17A, it can also act independently [37, 38]. Unlike many other T cell-produced cytokines, IL-22 does not act on immune cells. Rather, IL-22 mainly acts on epithelial cells and hepatocytes [38–40]. Its functions include antimicrobial defense, cell regeneration and protection against tissue damage. Like other cytokines, IL-22 has both pro-inflammatory and anti-inflammatory effects [41]. IL-22 acts directly on epithelial and some fibroblast cells by inducing an acute phase response and expression of chemokines, matrix metalloproteinases, and antimicrobial proteins [33, 34, 42–44]. In the context of *H*. *pylori* infection, there is not much data. In 2014, polymorphisms in *Il22* were found to be associated significantly with gastric MALT lymphoma in Taiwan [45]. This study demonstrated that when *H*. *pylori* patients had higher IL-22 expression they were more likely to respond to therapy. They also demonstrated that IL-22 expression increased expression of RegIIIα and LCN2.

In this study, we investigated the role of IL-17A and IL-22 in gastric epithelial cell defense against *H*. *pylori*. Our findings suggest that IL-17A and IL-22 can act synergistically to induce expression of several antimicrobials. While these data suggest that Th17 cytokines may enhance expression of antimicrobials and help control *H*. *pylori* infection, IL-22 deficiency alone did not alter control of *H*. *pylori* colonization and gastritis in the mouse model.

## Material and Methods

### Ethics statement

All animal experiments were performed in accordance with the Animal Welfare Act and U.S. federal law. All experiments were carried out at Vanderbilt University under protocol numbers V/10/410 and V/13/240 and were approved by the Department of Veteran's Affairs Committee and Vanderbilt University Institutional Animal Care and Use Committee (IACUC) which is accredited by the Association of Assessment and Accreditation of Laboratory Animal Care (AAALAC). All animals were housed under these guidelines in an accredited research animal facility fully staffed with trained personnel.

### Bacterial strains and growth conditions

This project employed the use of the Pre-mouse Sydney Strain 1 (PMSS1) of *Helicobacter pylori* or the mouse-passaged derivative, SS1. Bacteria were grown on Trypticase soy agar (TSA) plates containing 5% sheep blood. Alternatively, bacteria were grown in Brucella broth containing 10% heat-inactivated fetal bovine serum (FBS) and 10μg/ml vancomycin. Cultures were grown at 37°C either in an incubator supplemented with 5% CO_2_ or under microaerophilic conditions generated by a GasPak^™^ EZ Campy Container System (BD). Liquid cultures for infection were grown under microaerophilic conditions with shaking at 150 rpm. In order to isolate *H*. *pylori* from the stomachs of the mice, stomach homogenate was placed on TSA plates containing sheep blood (5%), nalidixic acid (10 μg/ml), vancomycin (50 μg/ml), amphotericin (2 μg/ml) and bacitracin (100 μg/ml) and cultured under microaerophilic conditions at 37°C for 3–5 days.

### Animals and experimental challenge

A breeding pair of interleukin-22 (UNQ3099) knockout mice (IL-22-/-; C57BL/6J background) was obtained from Genentech, Inc. in order to develop an experimental colony, while wild-type (C57BL/6J) mice were procured from The Jackson Laboratory. *Helicobacter*-free IL-22-/- and IL-22+/+ (wild-type) male littermates, 8 to 10 weeks old, were used in all experiments. The IL-22-/- breeding pairs tested negative for intestinal *Helicobacter*. Feces from sentinel mice housed in the same room consistently tested negative for pinworms, mouse parvovirus, and several other murine pathogens. In order to infect the mice for this project, *H*. *pylori* were inoculated into liquid medium and were cultured for 18 hours overnight and under microaerophilic conditions, as described above. Mice were then orogastrically inoculated with a suspension of 5×10^8^ (high dose) or 5×10^6^ (low dose) CFU of *H*. *pylori* in 0.5 ml of *Brucella* broth. Each dose was given twice, two days apart. The mice were then euthanized after 1 month or 3 months post infection and tissue collected for analyses.

### Harvest and stomach processing

The stomach was removed from each mouse by excising between the esophagus and the duodenum. The forestomach (nonglandular portion) was removed from the glandular stomach and discarded. The glandular stomach was opened, rinsed gently in cold PBS, and cut into three longitudinal strips that were used for bacterial culture, RNA analysis, and histology. For culturing *H*. *pylori* from the stomach, gastric tissue was placed into *Brucella* broth-10% FBS for immediate processing. Gastric tissue was stored in RNALater solution for subsequent RNA isolation at -20°C. A longitudinal strip from the greater curvature of the stomach was excised and placed in 10% normal buffered formalin for 24 hours, embedded in paraffin and processed routinely for hematoxylin and eosin (H&E) staining. Indices of inflammation were scored by a single pathologist (MBP) who was blinded to the identity of the mice. Acute and chronic inflammation in the gastric antrum and corpus were graded on a 0–3 scale. Acute inflammation was graded based on density of neutrophils and chronic inflammation was graded based on the density of lamina propria mononuclear cell infiltration. Total inflammation was calculated as a sum of acute and chronic inflammation in the corpus and the antrum allowing for quantification of total inflammation on a scale of 0–12.

### RNA extraction and real-time rtPCR

RNA was isolated using the TRIZOL isolation protocol (Invitrogen, Carlsbad, CA) with slight modifications, as previously described [21]. In the case of murine stomach tissue, the tissue was homogenized with gentleMACS™ Dissociator (Miltenyi Biotec, San Diego, CA). RNA was reverse transcribed using the High Capacity cDNA Reverse Transcription Kit (Applied Biosystems, Foster City, CA). For real-time rtPCR, we used the relative gene expression method. Glyceraldehyde 3-phosphate dehydrogenase (*Gapdh*) served as the normalizer, and tissue from uninfected WT mouse stomachs served as the calibrator sample. All real-time rtPCR was performed using an Applied Biosystems StepOne Plus real-time PCR instrument. Levels of gene transcription are indicated as “relative units”, based on comparison of tissue from *H*. *pylori*-infected mice with tissue from uninfected mice (calibrator tissue). In experiments on primary mouse epithelial cells and gastroids, levels of gene expression are indicated as “relative units”, based on comparison of stimulated cultures with unstimulated cultures (calibrator). Transcription of *DefB3*, *Lcn2* and *S100a9* were not always detected in the unstimulated cultures. When that was the case, the relative unit value was derived by assigning a C_T_ of 40 to the unstimulated sample. Primer and probe sets were purchased as Taqman Gene Expression Assays from Applied Biosystems (as pre-designed assays the annealing temperatures and amplicon length are available on their website) [mouse primer sets: *DefB3* (Mm01614469_m1), *Cxcl1* (Mm01354329_g1), *Cxcl2 (Mm00436450_m1)*, *Cxcl5* (Mm00436451), *Lcn2* (Mm01324473_g1), *S100a8* (Mm01220132), *S100a9* (Mm00656925_m1), *Il17a* (Mm00439619_m1), *Ifnγ* (Mm99999071_m1) and *Gapdh* (Mm99999915_g1); human primer sets: *defβ1* (*DEFB-1*) (Hs00174765_m1), *defβ4a* (*DEFB-4*) (Hs00175474_m1), *defβ103* (*DEFB-3*) (Hs00218678_m1), *IL-8* (Hs00174103_m1), *LCN2* (Hs00194353_m1), *S100A8* (Hs00374264_g1), *S100A9* (Hs00610058_m1) and *GAPDH* (Hs99999905_m1)].

### Culturing and stimulating mouse primary gastric cells

The stomach from WT mice was aseptically removed, opened and washed in sterile PBS in order to remove contents. Then the stomach was transferred to a solution of 0.04% bleach in PBS where it rested for 15 min at room temperature. Following that, the stomach was again rinsed in sterile PBS and then placed into a tube with 25 mL EDTA/DTT solution for incubation at room temperature for 1.5 hrs. The EDTA/DTT solution was poured off and 20 mL sterile PBS was added to the tube and then shaken vigorously until crypts came into solution indicated by the opaque appearance of the solution. The cell suspension was then filtered through a cell strainer to remove impurities and the supernatant was centrifuged at 1,500 RPM for 7 mins at room temperature. The supernatant was then discarded and the cell pellet was resuspended in 20 mL fresh PBS. Centrifugation was repeated and the pellet was resuspended in 1X culture media (Hams F-12 1X + 1/100 Penicillin and Streptomycin antibiotics, glutamine 1/100 and 5% FBS), that was previously incubated at 37°C. From 1 stomach, 4–4.5 mL suspension was obtained. Finally, cells were plated in collagen/laminin coated 48-well plates (0.5 mL/well) and were left to rest for at least 20 hrs. Next, the cells were stimulated with murine recombinant IL-17A and IL-22 cytokines (PeproTech). To do this, the media was removed from the cells and the cells were washed with PBS. Then 0.5mL of fresh 1X culture media containing 50 ng/mL rIL-17a and 200 ng/mL rIL-22 was added to each well with the cells (these concentrations were previously described, [44, 46]). Media with no cytokines was used as a control. Following stimulation, the media was discarded and cells were prepared for RNA extraction and real-time rtPCR.

### Gastroid generation, culture and stimulation

This protocol was carried out as previously described [47] with minor modifications. A stomach was tied and dissected from a 6–8 weeks old mouse and washed in cold DPBS (Gibco). The stomach was then opened at the fundus, inverted, tied and injected with DPBS to inflate. Following that, 5mM EDTA solution was added and the stomach left to rock gently in cold room for 2hrs. Then, the EDTA was removed and 5mL shaking buffer (DPBS, 54.9mM D-sorbitol and 43.4mM Sucrose) was added for shaking very slowly (less than 1 min intervals) by hand for 2 mins. A 20μL sample of the solution was then checked under a light microscope for glands (about 5–10). Next, 1mL samples were transferred to Eppendorf tubes and centrifuged at 300g for 10mins. The supernatant was then removed and matrigel containing growth factors was added. Samples were then added to 12 well plates, overlayed with minigut culture media and incubated at 37° for 3 days in 5%CO_2_. At the end of the three day incubation period, the gastroids were stimulated as were the primary cells described above. RNA extraction and rtPCR was also conducted as previously described.

### Cytokine treatment of epithelial cells

AGS (ATCC CRL-1739) or AZ-521 (JCRB0061; also known as HuTu-80 cells, duodenal origin), were washed with PBS and detached from the cell culture flask using Trypsin/EDTA treatment. After enumerating the cells, the epithelial cells were plated in a 24-well plate at 1x10^6^ cells/ml in serum-free RPMI (Gibco) with 300 μl/well. After 2 hours cells were treated with PBS, human rIL-17a (Peprotech, 50 ng/ml), human rIL-22 (Peprotech, 200 ng/ml) or both rhIL-17A and rhIL-22. These concentrations were chosen based on previous reports [44, 46]. Human epithelial cells were stimulated at several time points including 4 hr, 8 hr, 12 hr and 24 hr. At the designated time point, the cell supernatant was removed for IL-8 measurements (by ELISA) and cells were washed gently with 1X PBS. TRIzol^®^ Reagent (Ambion, 0.5mL/ well) was applied directly to the well and after pipetting up and down several times to lyse the cells, the TRIzol was collected and RNA was isolated.

### *H*. *pylori* killing assay

AGS or AZ-521 cells were stimulated with PBS, rhIL-17A (50 ng/ml), rhIL-22 (200 ng/ml), or both rhIL-17A and rhIL-22 for 16 hours. The following day, *H*. *pylori* (strain SS1, 60190 or PMSS1) were added to the wells containing epithelial cells at a multiplicity of infection (MOI) of 5 or 50. The MOI of infection was estimated based on OD_600_ but the input was then back calculated. The MOI reported is from the back calculation. The *H*. *pylori*–epithelial cell co-culture was incubated at 37°C/5% CO_2_ for 6 hours. The epithelial cell-mediated killing was assessed by plating serial dilutions of live *H*. *pylori* from each well (both nonadherent and adherent bacteria were collected) on TSA with 5% sheep blood. *H*. *pylori* CFU were enumerated after plates were incubated for 3 days at 37°C/5% CO_2_. The data are presented as percent survival. The percent survival was calculated by dividing [the number of CFU recovered from the co-culture *H*. *pylori* and cytokine stimulated epithelial cells] by [the number of CFU recovered from the co-culture of *H*. *pylori* with unstimulated epithelial cells]. Cytokine treatment of human epithelial cells did not affect epithelial cell survival or proliferation. From time 0 (plating the epithelial cells) to harvesting bacteria for determining *H*. *pylori* survival, human epithelial cell counts were similar under all conditions (media alone, media +IL-17A, media +IL-22, or media +IL-17A and IL-22 (data not shown). Moreover, *H*. *pylori* survival was not directly affected by cytokines in the absence of human epithelial cells (data not shown).

## Results

### Human gastrointestinal epithelial cells mount an antimicrobial response to *H*. *pylori* in the presence of Th17 cytokines

**Fig 1:**
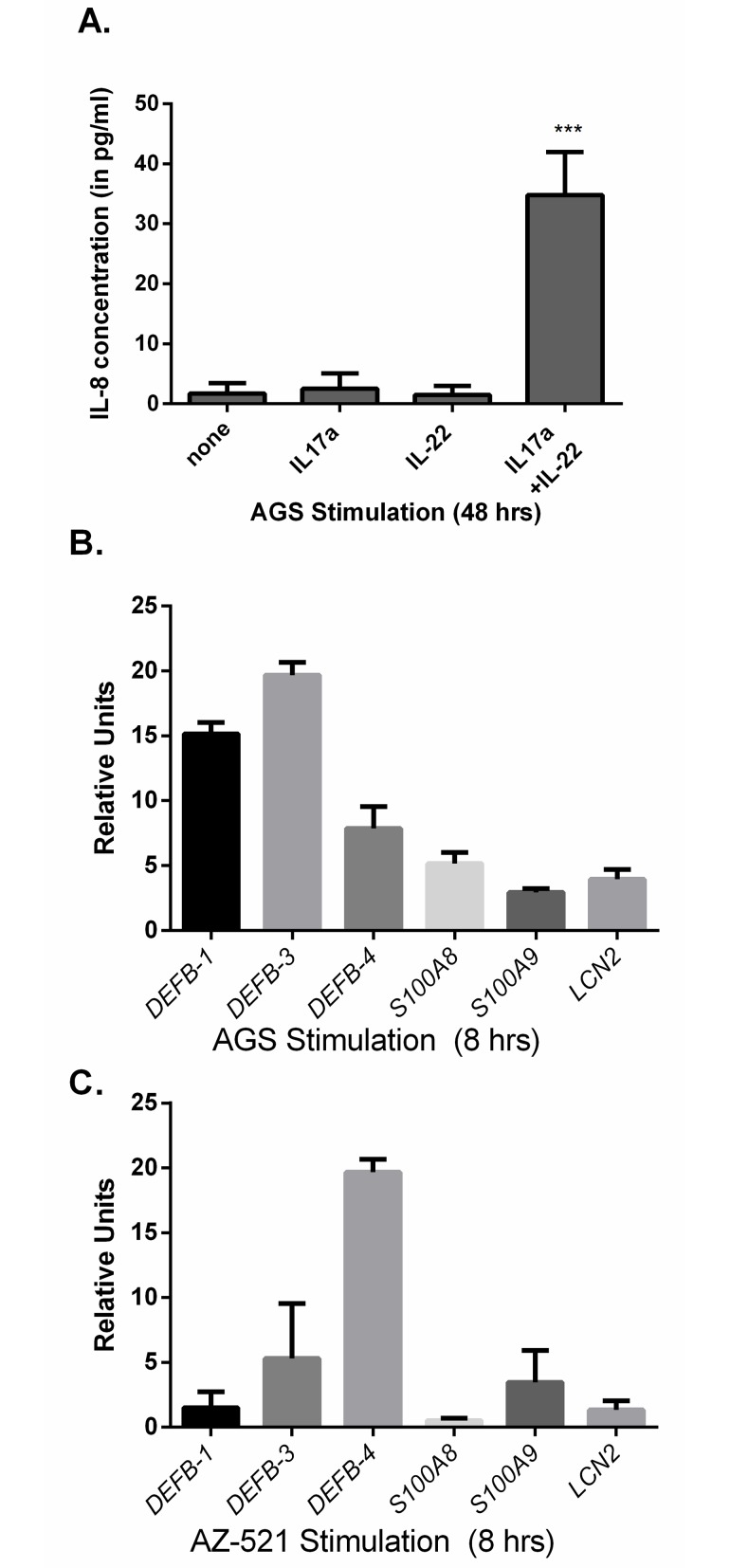
IL-17A and IL-22 synergistically stimulated human epithelial cells inducing IL-8. (A) IL-8 production at 24 hours by AGS cells in response to IL-22 and IL-17A as measured by ELISA. p-value is based on ANOVA test with Dunnett’s correction for multiple comparisons ***p<0.001. (B) 8 hour stimulation of AGS cells or (C) AZ-521 cells with an IL-22/IL-17A cocktail induces upregulation of antimicrobials as measured by real-time rtPCR. Expression is shown as relative units and is relative to RNA from untreated cells. Data shown as ± SEM and are representative of 3 independent experiments.

AGS cells were stimulated with recombinant cytokines in order to determine if human gastric epithelial cells produce neutrophil recruiting chemokine, IL-8, and antimicrobials in response to the Th17 cytokines IL-17A and IL-22. After stimulating AGS cells with IL-17A (50 ng/ml), IL-22 (200 ng/ml) or with both IL-17a and IL-22 for 24 hrs, the concentration of IL-8 was measured by ELISA. IL-17A and IL-22 synergistically stimulated human epithelial cells to induce IL-8, while stimulation of IL-17A or IL-22 alone did not significantly induce IL-8 (Fig 1A).

To assess antimicrobial gene expression, AGS or AZ-521 cells were stimulated with IL-17A, IL-22 or both IL-17A and IL-22 for 8 hours and the gene expression of several antimicrobials were measured by real-time rtPCR. The response to IL-17A or IL-22 alone was minimal (S1 Fig), but IL-17A and IL-22 together induced an amplified response and expression of antimicrobials including *DEFB-1*, *DEFB-3*, *DEFB-4 S100A8*, *S100A9*, *and LCN2*. (Fig 1B and 1C). There were notable differences in the amplification of the antimicrobial genes in stimulated AGS cells and stimulated AZ-521 cells. The antimicrobials were less amplified in the AZ-521 cells than AGS cells with the exception of *DEFB-4* in AZ-521 with its amplification a little under four times that of AGS cells. One potential explanation for differences in expression of antimicrobials in these cell lines is that they are derived from different areas of the gastrointestinal tract that may express varying levels of cytokine receptors on their surface.

**Fig 2:**
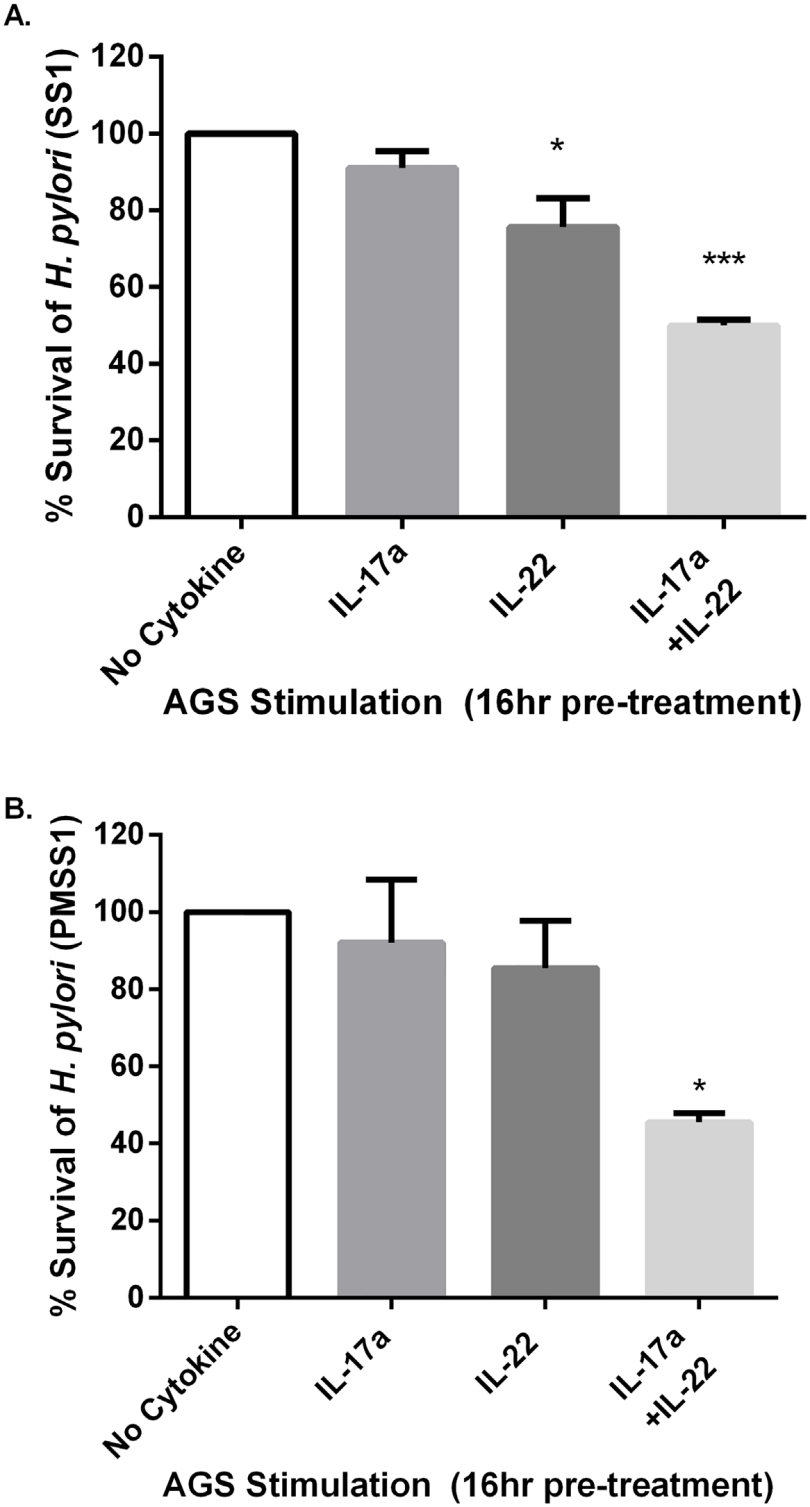
IL-17A and IL-22 can activate human gastrointestinal epithelial cells to kill *H*. *pylori*. The ability of *H*. *pylori* to survive for 6 hours in co-culture with AGS cells pre-stimulated for 16 hours with IL-22, IL-17A or combined IL-22 and IL-17A is presented. (A) SS1 strain or (B) PMSS1 strain percent (%) survival represented on the y-axis is equal to the number of *H*. *pylori* CFU from the cytokine-treated cells divided by the number of CFU recovered from untreated AGS cells. Graphs are representative of 3 independent experiments and error bars represented mean ± SEM. Statistical significance is based on one-way ANOVA test with Dunnett’s correction for multiple comparisons to the no cytokine treatment control ***p<0.001,**p<0.01,*p<0.05.

To ascertain whether or not this response had an effect on bacterial growth or survival, an investigation was conducted using an epithelial cell—*H*. *pylori* co-culture model killing assay. AGS cells or AZ-521 cells were stimulated with IL-17A, IL-22, both IL-17A and IL-22, or left unstimulated. After 16 hours of culture, *H*. *pylori* were added at an MOI of 5 or 50. The ability of *H*. *pylori* to survive was assessed 6 hours later by plating serial dilutions of the co-culture. Epithelial cells stimulated with both IL-17A and IL-22 killed the most *H*. *pylori* (Fig 2, S1C Fig). Direct addition of IL-17A and IL-22 to *H*. *pylori* did not affect *H*. *pylori* growth in the absence of epithelial cells (data not shown). Moreover, stimulation of AGS cells with IL-17A and IL-22 did not affect epithelial cell survival over 24 hours. At an MOI of 5 and 50 with *H*. *pylori* AGS cells were still viable (>95%) after 6 hours of co-culture (data not shown). These data indicate that IL-17A and IL-22 synergistically activate an effective antimicrobial response to *H*. *pylori* in AGS cells.

### Antimicrobial response to IL-22 and IL-17A in murine epithelial cells and gastroids

In order to analyze the effect of the Th17 cytokines IL-22 and IL-17A on murine gastric epithelia cell responses, mouse stomach epithelial cells were treated with both recombinant IL-17A and IL-22 or with IL-22 alone. The gene expression levels of *Cxcl1*, *Cxcl5*, *Lcn2* and *S100a8* were measured after 8 hours of stimulation. Expression of *Cxcl1* and *Cxcl5*, functional homologs of IL-8, a neutrophil attracting chemokine, was measured as a control for GEC responsiveness. Our previously published work describes primary mouse gastric epithelial cells response to IL-17A leads to increased gene expression of these chemokines [24]. The results were normalized to unstimulated cells grown in media alone. As expected, the expression levels of *Cxcl1* also increased 8 hours after stimulation with both IL-17A and IL-22 (S2A Fig). This may be one mechanism by which infiltration of neutrophils is mediated in the lamina propria during chronic gastric inflammation. However, stimulation with IL-22 alone did not change the transcript levels of either *Cxcl1* or *Cxcl5*. Transcription of *S100a8* modestly increased upon cytokine stimulation, and while the gene expression of lipocalin 2 (*Lcn2*) was moderately upregulated in some experiments the results were not consistent (data not shown).

Due to the limitations of working with primary gastric epithelial cell preparations, including few cells and difficulty to keep these cells alive for more than 48 hours, the gastroid model was used. Gastroids are three-dimensional model systems that replicate events in the gastric epithelium more closely than traditional clonal cell culture systems [46]. Thus, they provide an ideal opportunity for the molecular dissection of epithelial responses to cytokines. Wroblewski, *et al*. established this notion by demonstrating that murine gastroids self-organize into a sphere of cells that surround a central lumen and differentiate into mucus cells, parietal cells, G-cells, enterochromaffin-like (ECL) cells and D-cells [46].

For our purposes, gastroids were generated from WT mouse stomachs and cultured in matrigel with appropriate growth factors, as described in the materials and methods. After 3 days, the gastroids were stimulated, as were the murine epithelial cells, with a cocktail of recombinant murine IL-22 and IL-17a for 8 hours. Upon cytokine stimulation, rtPCR was conducted to assess levels of antimicrobial gene transcription. Juxtapose to the results found in the Th17 cytokine stimulation of murine epithelial cells, it was observed that *Cxcl1*, *Cxcl5*, *Defb3*, *Lcn2*, *S100a8* and *S100a9* were upregulated when compared to unstimulated gastroids (S2B Fig). On the other hand, although *Cxcl2* and *Defb2* were investigated, they were neither detected in the unstimulated nor the stimulated gastroids.

### IL-22 expression is induced during *H*. *pylori* infection

**Fig 3:**
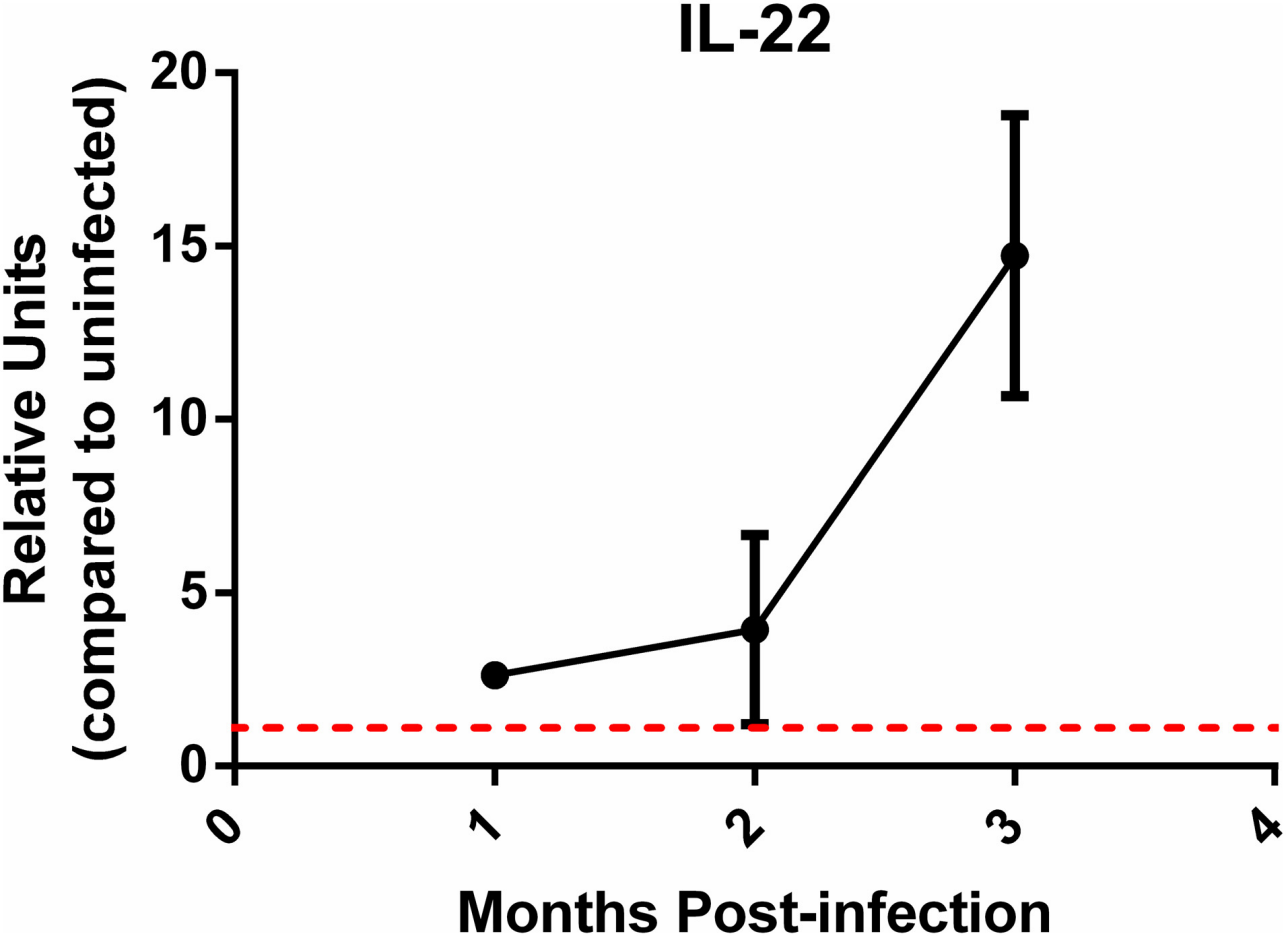
*H*. *pylori* infection induces IL-22 expression in murine gastric mucosa. Expression of IL-22 in *H*. *pylori*-infected WT mice at 1, 2 and 3 months post infection was determined by real-time rtPCR. Relative units (relative to uninfected WT mice) is presented as the mean of 4–6 WT mice at each time point. Error bars represent ± SEM.

Expression of IL-22 has been reported in the *H*. *pylori*-infected gastric tissue of humans [27] and gastric cancer patients [28, 29]. To address whether IL-22 expression is increased in the mouse model of infection, gastric tissue was collected from infected and uninfected mice over time. Expression of IL-22 in *H*. *pylori*-infected WT mice at 1 month, 2 months and 3 months post infection was measured by real-time rtPCR (Fig 3). *H*. *pylori* infection with PMSS1 induced IL-22 expression in murine gastric mucosa.

### IL-22 deficient mice control *H*. *pylori* colonization

**Fig 4:**
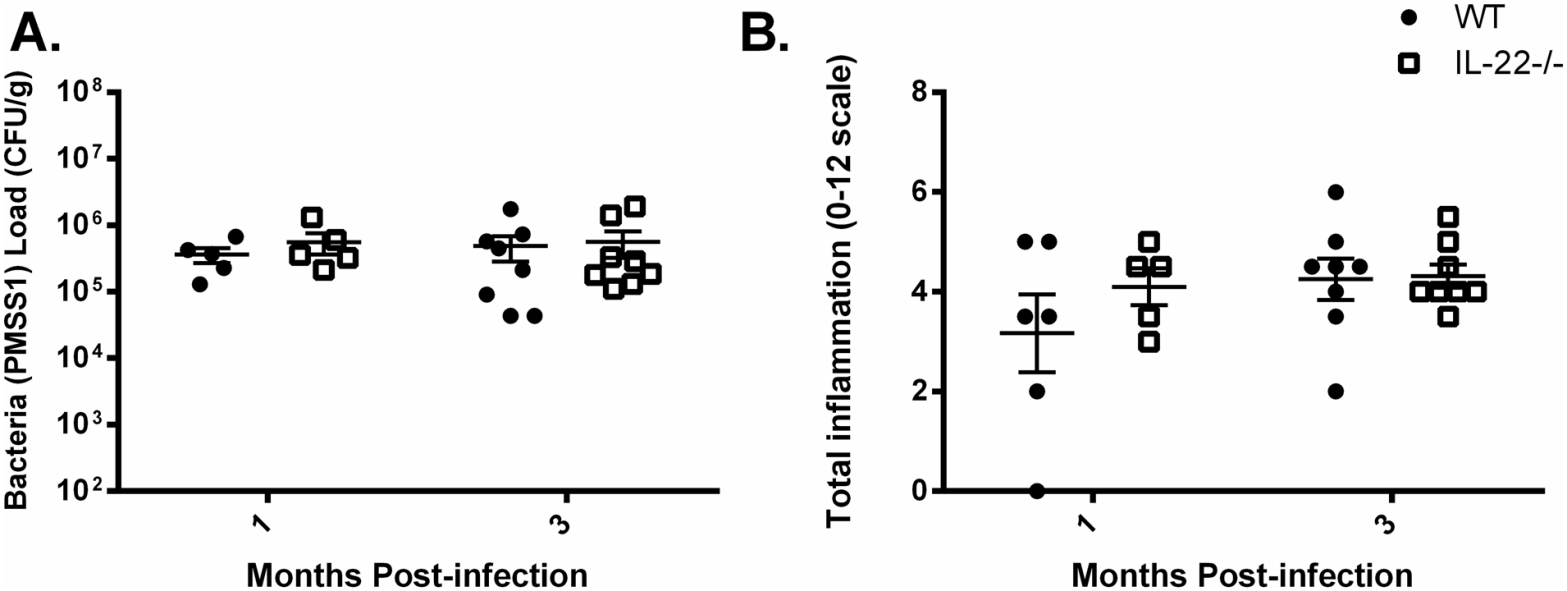
IL-22 is not required to control bacterial burden or gastric inflammation in the mouse model. (A) Bacterial burden was measured in WT and IL-22-/- mice that were infected with PMSS1 for 1 month or 3 months. Colony forming units (CFU) per gram of stomach tissues was calculated and is presented in the graph. (B) Levels of acute and chronic inflammation were scored on stomach tissue (in the corpus and antrum) at 1 month and 3 months post infection with strain PMSS1. Total inflammation as presented is the sum of acute and chronic inflammation. Statistical analysis was performed using Mann-Whitney U which resulted in no significant differences between the groups, and error bars represented mean ± SEM. See methods for scoring system (scale is 0–12).

Since IL-22 works with IL-17A to stimulate the strongest antimicrobial response in gastric epithelial cells of humans and of mice, the importance of IL-22 alone was investigated in a mouse model of gastritis. IL-22-deficient mice and WT C57BL/6 mice were infected with *H*. *pylori* strain PMSS1, a clinically relevant strain which has a functional *cag*-type IV secretion system, or SS1 (a mouse-passaged strain of PMSS1) which no longer has a functional *cag*-type IV secretion system. These mice received two orogastric doses (5x10^8^ CFU/dose of *H*. *pylori*). The ability of the mice to control *H*. *pylori* colonization was assessed by determining bacterial burden in the gastric tissue. Time points up to 3 months post infection (Fig 4A) indicate that IL-22-deficient mice are able to control *H*. *pylori* colonization just as well as WT mice. Similar results were observed when mice were infected with the SS1 strain (S3 Fig).

### *H*. *pylori*-infected IL-22-deficient mice develop gastritis similar to WT mice

It has been demonstrated that IL-22 not only plays a role in induction of antimicrobial responses but is important for barrier function and may have an anti-inflammatory role in the gastric mucosa through regulation of protective mediators such as follistatin, IL-10, and IL-11 [48, 49]. To determine whether IL-22 has a role in the inflammatory response to *H*. *pylori*, the level of inflammation was quantified in *H*. *pylori*-infected IL-22-deficient mice and WT mice in both regular dose and low dose experiments. IL-22-deficient mice and WT mice had similar levels of acute and chronic inflammation as scored from H&E staining of the gastric tissue at 1 month post infection (Fig 4B, S2B Fig).

### Low dose infection of WT and IL-22-/- mice

The dose of infection used in the chronic experiment (Fig 4) is based on a model of gastritis, meaning the 2 high doses of *H*. *pylori* are sufficient to induce gastritis in the mouse model. Since the experimental design for this study was not to induce gastritis but to investigate an antimicrobial response, the experiment was repeated with a lower dose. IL-22-deficient mice and WT controls were infected with *H*. *pylori* at a dose of 5x10^6^ CFU/mL (low dose) and 5x10^8^ CFU/mL (high dose). In this experiment, even with the low dose of infection, the IL-22-deficient mice had lower bacterial burdens in their gastric tissue at 4 weeks post infection compared to WT mice (S4A Fig). This was unexpected if IL-22 has a role in activating antimicrobial responses. Moreover, there is no significant difference in inflammation between infected WT and infected IL-22 deficient mice during this infection (S4B Fig). Therefore, in the mouse model IL-22 deficiency does not render mice more susceptible to *H*. *pylori*.

### Expression of several antimicrobials are unaffected by IL-22 deficiency

**Fig 5:**
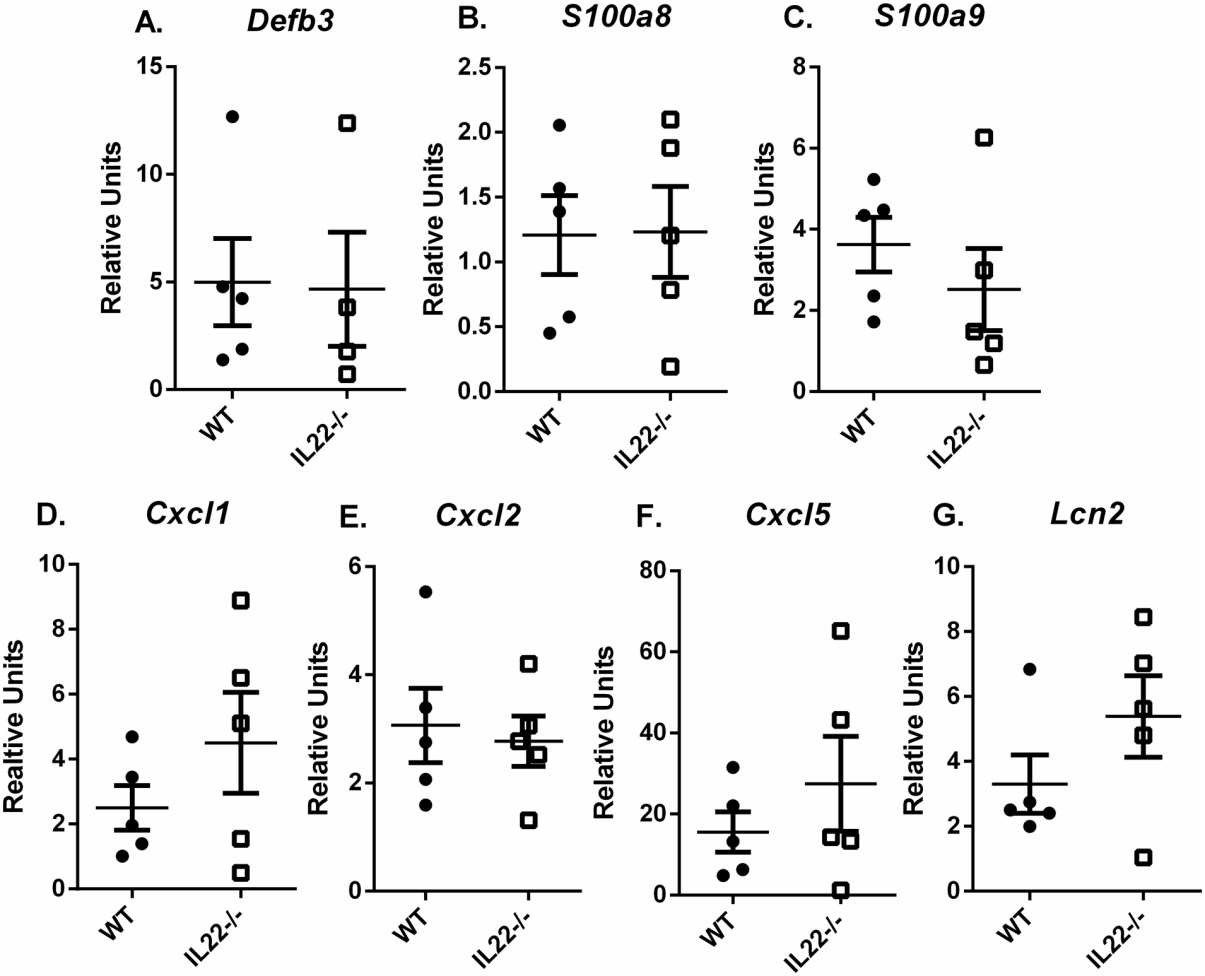
IL-22 deficiency had minimal effect on gene expression in the mouse during *H*. *pylori* infection. At 1 months post infection, no significant difference in expression of *Defb3* (p = 0.524), *Lcn2* (p = 0.31), *S100a8* (p = 0.944), *S100a9* (p = 0.31), *Cxcl1* (p = 0.413), *Cxcl2* (p = 0.944) and *Cxcl5* (p = 0.532) as measured between *H*. *pylori*–infected WT and infected IL-22-/- mice gastric tissue. Relative units are calculated as described in the methods, relative to GAPDH and calibrated to uninfected WT mice. Statistical analysis was performed using Mann-Whitney U (Error bars represent ± SEM).

To determine if the loss of IL-22 had an effect on antimicrobial gene expression, the levels of expression were determined at 1 month post infection after high dose or low dose infection. At 1 month post infection, RNA was isolated from the gastric tissue of *H*. *pylori*-infected WT and IL-22-deficient mice. Expression of *Defb3*, *Lcn2*, *S100a8*, *S100a9*, and IL-8 homologs (*Cxcl1*, *Cxcl2 and Cxcl5*) was measured by real-time rtPCR. IL-22 deficiency had minimal effect on antimicrobial gene expression, chemokine gene expression (Fig 5), or Ifnγ and Il17a levels (data not shown) in the mouse during *H*. *pylori* infection.

## Discussion

The role of IL-22 at mucosal surfaces has been investigated in many models. Its role has been described primarily as barrier defense and wound healing [50–52]. It acts through a receptor, a heterodimer of IL-22R1 and IL10R2, which is present primarily on non-hematopoietic cells of the skin, respiratory and digestive tissues [53]. The goal of this study was to determine if IL-22 plays a role in activation of antimicrobial responses and control of *H*. *pylori* colonization. Our data indicate that IL-22 alone does not activate antimicrobial responses, but that IL-22 synergizes well with IL-17A for these effects. Moreover, IL-22 deficiency alone does not increase mouse susceptibility to *H*. *pylori* infection. Finally, our data suggest that IL-22 is not required for the pro-inflammatory gastritis response to *H*. *pylori* infection. In one experiment (S3 Fig) we observed a decrease in bacterial burden in IL-22-/- mice compared to WT mice, but no significant difference in inflammation scores. This may be due to a change in the barrier function of the stomach, but the data only further support the conclusion that IL-22 alone does not play a role in control of bacterial colonization through induction of antimicrobials.

While antimicrobial responses have been shown to be vital for control of pathogens in the gastrointestinal tract, very little is known about which antimicrobials play a role in control of *H*. *pylori* infection. Our study (Fig 1) and others have demonstrated that IL-22 can play a role in expression of S100A8 and S100A9 [27, 34, 54]. While the S100A8 and S100A9 proteins have been characterized as antimicrobial, their role during *H*. *pylori* infection may be multi-dimensional [55]. S100A8 and S100A9 form a heterodimer known as calprotectin, which can affect growth or virulence of *H*. *pylori* through sequestration of nutrient metals, especially zinc [55]. Furthermore, calprotectin has also been linked to inflammation through ligation of TLR4 and receptor for advanced glycation end products (RAGE) [56–58]. For this reason it has been called a Damage Associated Molecular Pattern or DAMP. Therefore, differentiating between the functions of calprotectin, an antimicrobial of the nutritional immune response and a pro-inflammatory DAMP, is challenging. Nevertheless, a significant number of studies have been done to extrapolate the antimicrobial function of the heterodimer [55, 59–61].

The IL-22 cytokine is required for elimination of the intestinal Gram-negative bacterium *Citrobacter rodentium* [62, 63]. IL-22 also induces Reg family proteins which may be vital for control of this bacterium [62, 64]. On the other hand, IL-22 is negligible for the host defense to *Mycobacterium tuberculosis* and *Listeria monocytogenes* infection [65, 66]. These findings are more similar to our model of *H*. *pylori* infection where IL-22 seems to be dispensable for control of infection and for development of gastritis. However, animal experiments demonstrate that IL-22 is also important for the host defense to *M*. *avium* and *Salmonella enterica* [67–69]. These data are evidence that IL-22 can act as a proinflammatory or anti-inflammatory cytokine depending on the inflammatory context.

Bronchial epithelium and skin keratinocytes respond synergistically to IL-17a, IL-17f and IL-22 to induce many antimicrobials including β-defensin 2 and β-defensin 3 [31, 46]. Murine tracheal epithelial cells also respond synergistically to IL-17A and IL-22 to induce lipocalin-2 [31]. While CCL20 expression was increased in some studies with IL-17A alone [70, 71], more recent data indicate that IL-22 may downregulate CCL20 expression in gastric epithelial cells [27, 72] suggesting an anti-inflammatory role in this capacity.

Interleukin 22 was first described as a T cell-derived cytokine. Its expression was originally recognized as being associated with Th17 responses, and since that time has also been attributed to Th22 cells and innate lymphoid cells (ILCs) [36, 63, 73]. In a recent paper by Zhuang, *et al*., the authors suggested that Th22 cells contribute IL-22 during *H*. *pylori* infection [27], but they did not rule out Th17 cells. They attributed expression to IL-23, but they did not investigate whether the T cells expressing IL-22 are dependent on IL-6 or co-express IL-17A which would solidify their claim.

We, and others, have shown that IL-22 expression correlates with increased gastritis in the *H*. *pylori*-infected humans and *H*. *pylori*-infected mice [27]. Since IL-22 expression follows the same pattern as other pro-inflammatory T cell-derived cytokines, it is possible that this correlation is due to increased expression of a group of cytokines and not direct evidence that IL-22 drives inflammation. Zhuang *et al* recently described a regulatory network which requires IL-22 to exert its pro-inflammatory effects during *H*. *pylori* infection [27]. These data conflict with our mouse model studies. The major difference in our findings and those of the Zhuang paper is the background of the mouse model. In the Zhuang paper, the Balb/c WT mouse and the IL-22-/- mouse in the Balb/c background are used for their studies making it difficult to compare the studies since Balb/c and C57BL/6 mice, which we used, have differences in how their cytokines respond. It is possible that in the C57BL/6 mice other pro-inflammatory cytokines, such as IFNγ and IL-21 drive inflammation which may overcome the need for IL-22. Levels of IFNγ are not significantly different in IL-22-/- and WT mice in our *in vivo* model (data not shown). Additionally, another difference in this study was the use of PMSS1 and SS1 compared to their use of *H*. *pylori* strain NCTC 11637 (also a *cag*+ strain) [27]. The synergistic effects observed with IL-17A and IL-22 were previously observed in bronchial epithelial cells and skin [44, 46]. These effects and increased expression of IL-8 when both IL-22 and IL-17A are used for stimulation could be due to increased activation of P-Stat3. Since several proinflammatory cytokines including IL-21, IL-22, IL-23, IL-17, and TNF can activate P-Stat3 [74]. Another possibility is that IL-22 or IL-17A enhances cytokine receptor expression and enhances activation of epithelial cells. Loss of IL-22 may be compensated *in vivo* by the expression of these other pro-inflammatory cytokines.

These studies do bring to light some of the challenges of working with a human pathogen in mouse models. Unfortunately, working with human tissues and recovering live immune cells from stomach biopsies is not feasible in most populations. In 2014, polymorphisms in *Il22* were found to be associated significantly with gastric MALT lymphoma in Taiwan [45]. This study demonstrated that when patients had higher IL-22 expression they were more likely to respond to therapy. They also demonstrated that IL-22 expression increased expression of RegIIIα and LCN2. While our studies in the C57BL/6 mice demonstrate that IL-22 is dispensable for control of *H*. *pylori* infection, the data do demonstrate that *in vitro* IL-22 and IL-17a act synergistically to activate pro-inflammatory chemokine expression and antimicrobial transcription in human gastric epithelial cells.

## Supporting Information

S1 Fig Antimicrobial expression in human epithelial cells stimulated with IL-17A or IL-22. (A) 8 hour stimulation of AGS cells or (B) AZ-521 cells with either IL-17A or IL-22. Transcription of antimicrobials was measured by real-time rtPCR. Expression is shown as relative units and is relative to RNA from unstimulated cells. Data shown as ± SEM and are representative of 3 independent experiments. (C) The ability of SS1 strain *H*. *pylori* (MOI of 50) to survive for 6 hours in co-culture with AZ-521 cells pre-stimulated for 16 hours with IL-22, IL-17A or combined IL-22 and IL-17A is presented. Percent (%) survival represented on the y-axis is equal to the number of *H*. *pylori* CFU from the cytokine-treated cells divided by the number of CFU recovered from untreated AZ-521 cells. Graphs are representative of 3 independent experiments and error bars represented mean + SEM. Statistical significance is based on one-way ANOVA test with Dunnett’s correction for multiple comparisons to the no cytokine treatment control, p<0.01,*p<0.05.
(TIF)

S2 Fig IL-17A and IL-22 activate mouse primary gastric cells. (A) Primary gastric epithelial cells from WT mice were stimulated with rIL-17A and rIL-22 for 8 hours. Expression of antimicrobial genes is presented as relative units and is comparative to *Gapdh* (endogenous control) and calibrated to unstimulated gastric epithelial cells. Error bars represent ± SEM (B) Gastroids stimulated with rIL-17A and rIL-22 for 8 hours. Antimicrobial genes are presented as relative units expressed in comparison to *Gadph* and calibrated to unstimulated gastroids. Data represents 5 experiments and error bars represent ± SEM.
(TIF)

S3 Fig IL-22 is not required to control bacterial burden or gastric inflammation in the mouse model with SS1 infection. (A) There is no significant difference in bacterial burden observed between WT and IL-22-/- mice that were infected with SS1 for 1 month (*p* = 0.413) or 3 months (*p* = 0.683). Colony forming units (CFU) per gram of stomach tissues was calculated and is presented in the graph. Statistical analysis was performed on log transformed values using the Student’s unpaired T test. (B) Total inflammation observed in mice that were infected for 1 month (*p* = 0.952) or 3 months (*p* = 0.214). See methods for scoring system (scale is 0–12). Statistical analysis was performed using Mann-Whitney U (Error bars represent ± SEM in both panels).
(TIF)

S4 Fig A lower dose changed control of bacterial burden, but not inflammation in IL-22-/- mice. (A) Bacterial burden expressed as the CFU/gram of stomach in mice (WT or IL-22-/-) infected for 1 month with either a low dose (L) or a higher dose (H) of PMSS1; p-values were based on an unpaired t-test comparing infected WT to infected IL-22-/- mice log transformed CFU/g values. For low dose *p = 0.0186, and *p = 0.0348 for high dose. (B) Inflammation scores in mice infected with different doses of PMSS1. Scores are on a scale of 0–12. Error bars represent ± SEM.
(TIF)
