# Supplementary figures and images for: IL-17a and IL-22 Induce Expression of Antimicrobials in Gastrointestinal Epithelial Cells and May Contribute to Epithelial Cell Defense against Helicobacter pylori
Source: PLoS One. 2016 Feb 11;11(2):e0148514. doi: 10.1371/journal.pone.0148514 (PMC4750979; doi:10.1371/journal.pone.0148514)

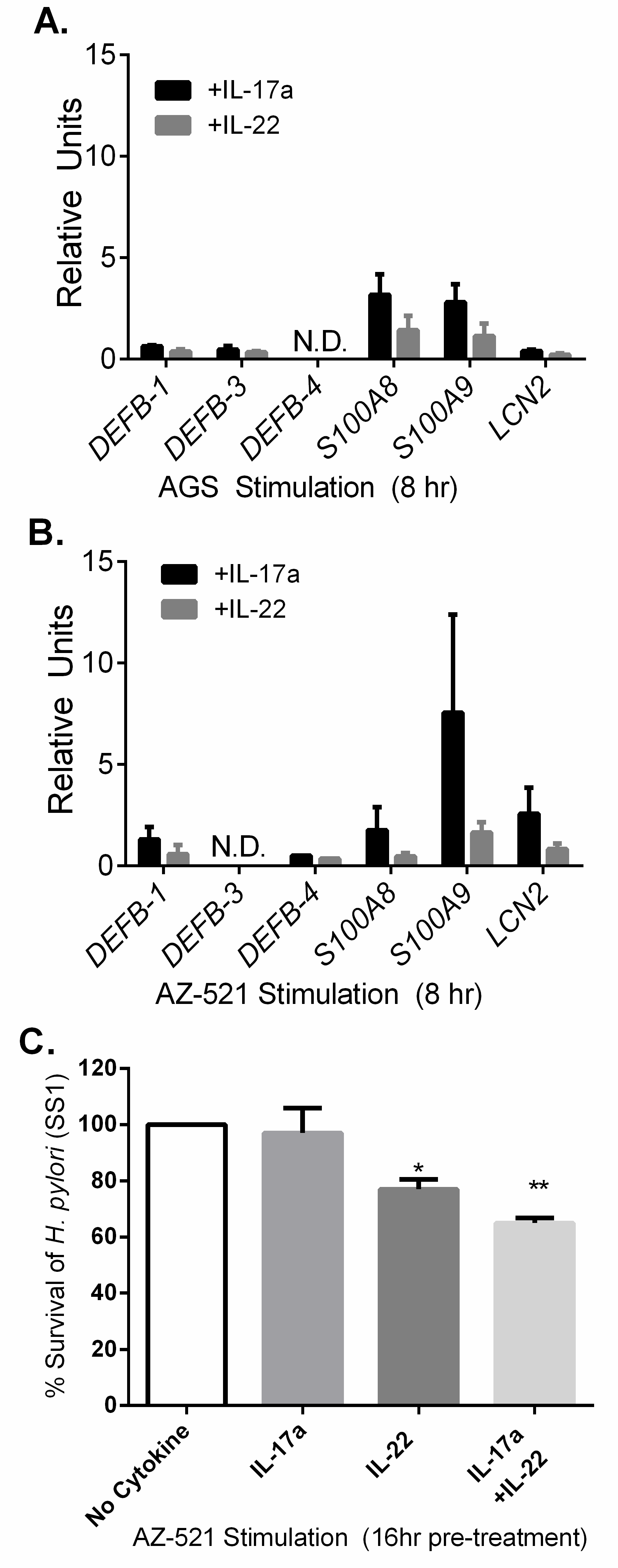

Supplement: S1 Fig — (A) 8 hour stimulation of AGS cells or (B) AZ-521 cells with either IL-17A or IL-22. Transcription of antimicrobials was measured by real-time rtPCR. Expression is shown as relative units and is relative to RNA from unstimulated cells. Data shown as ± SEM and are representative of 3 independent experiments. (C) The ability of SS1 strain H. pylori (MOI of 50) to survive for 6 hours in co-culture with AZ-521 cells pre-stimulated for 16 hours with IL-22, IL-17A or combined IL-22 and IL-17A is presented. Percent (%) survival represented on the y-axis is equal to the number of H. pylori CFU from the cytokine-treated cells divided by the number of CFU recovered from untreated AZ-521 cells. Graphs are representative of 3 independent experiments and error bars represented mean + SEM. Statistical significance is based on one-way ANOVA test with Dunnett’s correction for multiple comparisons to the no cytokine treatment control, p<0.01,*p<0.05. (TIF) [file pone.0148514.s001.tif]

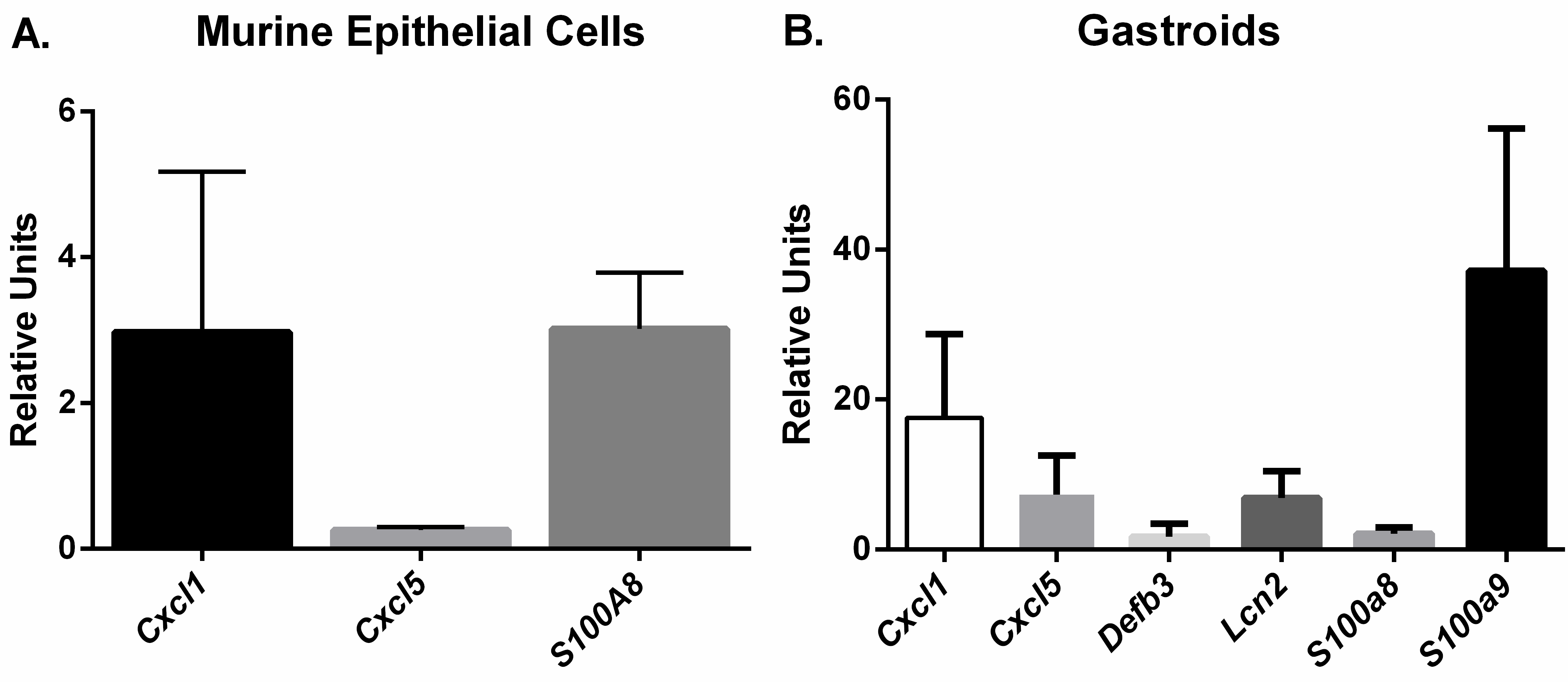

Supplement: S2 Fig — (A) Primary gastric epithelial cells from WT mice were stimulated with rIL-17A and rIL-22 for 8 hours. Expression of antimicrobial genes is presented as relative units and is comparative to Gapdh (endogenous control) and calibrated to unstimulated gastric epithelial cells. Error bars represent ± SEM (B) Gastroids stimulated with rIL-17A and rIL-22 for 8 hours. Antimicrobial genes are presented as relative units expressed in comparison to Gadph and calibrated to unstimulated gastroids. Data represents 5 experiments and error bars represent ± SEM. (TIF) [file pone.0148514.s002.tif]

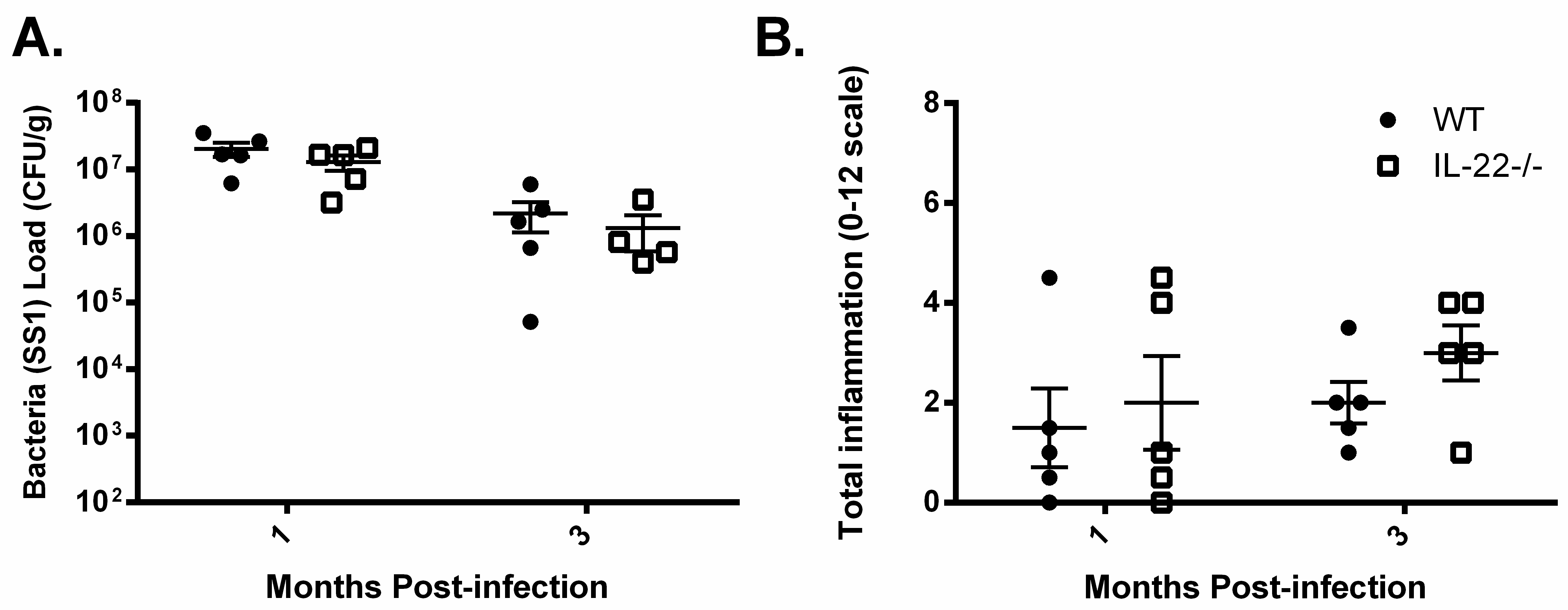

Supplement: S3 Fig — (A) There is no significant difference in bacterial burden observed between WT and IL-22-/- mice that were infected with SS1 for 1 month (p = 0.413) or 3 months (p = 0.683). Colony forming units (CFU) per gram of stomach tissues was calculated and is presented in the graph. Statistical analysis was performed on log transformed values using the Student’s unpaired T test. (B) Total inflammation observed in mice that were infected for 1 month (p = 0.952) or 3 months (p = 0.214). See methods for scoring system (scale is 0–12). Statistical analysis was performed using Mann-Whitney U (Error bars represent ± SEM in both panels). (TIF) [file pone.0148514.s003.tif]

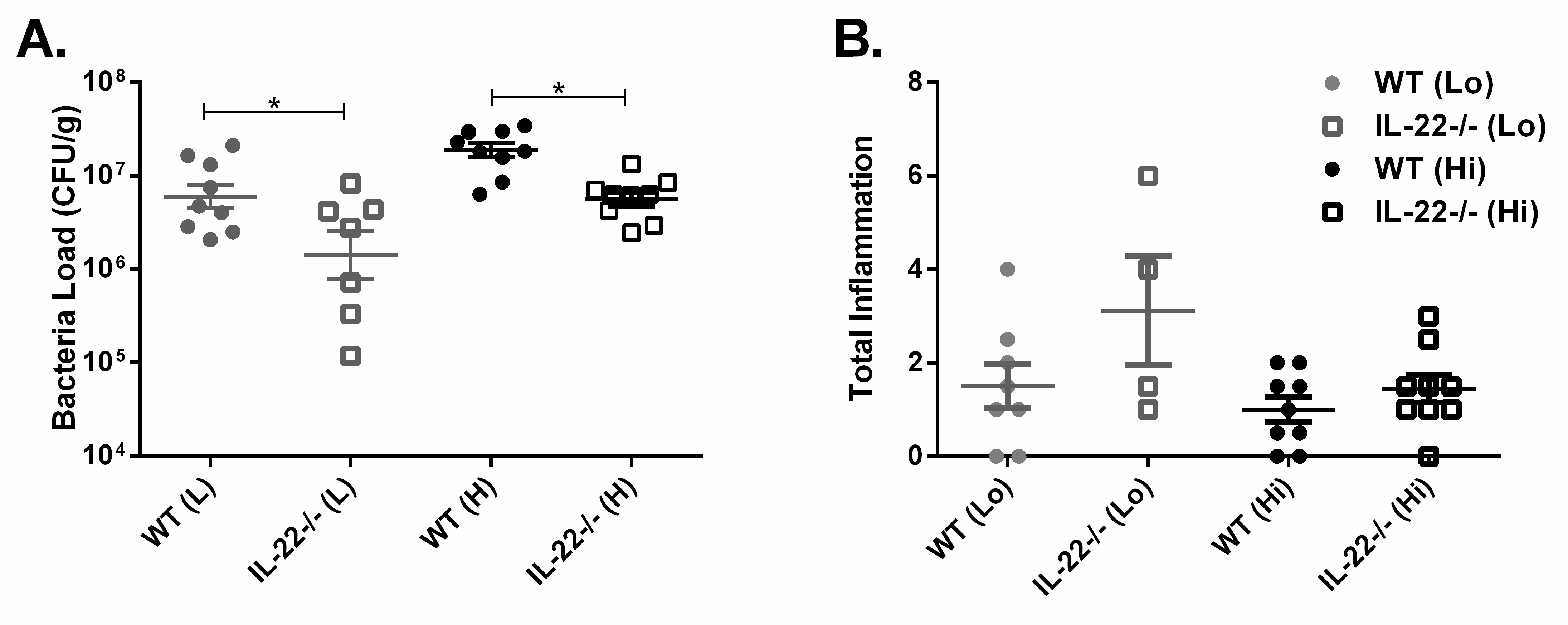

Supplement: S4 Fig — (A) Bacterial burden expressed as the CFU/gram of stomach in mice (WT or IL-22-/-) infected for 1 month with either a low dose (L) or a higher dose (H) of PMSS1; p-values were based on an unpaired t-test comparing infected WT to infected IL-22-/- mice log transformed CFU/g values. For low dose *p = 0.0186, and *p = 0.0348 for high dose. (B) Inflammation scores in mice infected with different doses of PMSS1. Scores are on a scale of 0–12. Error bars represent ± SEM. (TIF) [file pone.0148514.s004.tif]
